# Probing molecular dynamics at the nanoscale via an individual paramagnetic centre

**DOI:** 10.1038/ncomms9527

**Published:** 2015-10-12

**Authors:** T. Staudacher, N. Raatz, S. Pezzagna, J. Meijer, F. Reinhard, C. A. Meriles, J. Wrachtrup

**Affiliations:** 1 3rd Physics Institute, University of Stuttgart, 70569 Stuttgart, Germany; 2 IQST, University of Stuttgart, 70569 Stuttgart, Germany; 3 Max Planck Institute for Solid State Research, 70174 Stuttgart, Germany; 4 Department of Nuclear Solid State Physics, Institute for Experimental Physics II, University of Leipzig, Linnéstrasse 5, 04103 Leipzig, Germany; 5 Walter Schottky Institut and Department of Physics, TUM—Technical University of Munich, Am Coulombwall 4, 85748 Garching, Germany; 6 Department of Physics, CUNY—City College of New York, 160 Convent Avenue, New York, NY 10031, USA

## Abstract

We demonstrate a protocol using individual nitrogen-vacancy centres in diamond to observe the time evolution of proton spins from organic molecules located a few nanometres from the diamond surface. The protocol records temporal correlations among the interacting protons, and thus is sensitive to the local dynamics via its impact on the nuclear spin relaxation and interaction with the nitrogen vacancy. We gather information on the nanoscale rotational and translational diffusion dynamics by analysing the time dependence of the nuclear magnetic resonance signal. Applying this technique to liquid and solid samples, we find evidence that liquid samples form a semi-solid layer of 1.5-nm thickness on the surface of diamond, where translational diffusion is suppressed while rotational diffusion remains present. Extensions of the present technique could be exploited to highlight the chemical composition of molecules tethered to the diamond surface or to investigate thermally or chemically activated dynamical processes such as molecular folding.

## Abstract (web summary)

Nitrogen vacancy centres in diamond form spin-1 defects which may be exploited as sensitive probes of nanoscale magnetic phenomena. Here, the authors use individual nitrogen vacancies to perform nuclear magnetic resonance measurements of the ^1^H spins of adsorbed organic molecules.

## Graphical abstract

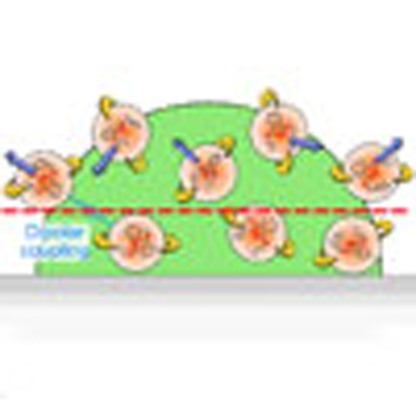

Nuclear magnetic resonance (NMR) is among the most versatile tools for investigating the dynamics of molecular processes down to the atomic level. It is widely used in the physical and life sciences, but has been limited to large sample quantities due to the low sensitivity of conventional detection methods^1^. Performing NMR detection at the nanoscale can substantially expand the microscopist's toolbox, potentially allowing for non-destructive imaging of complex macromolecules and/or studying the dynamics of diverse biochemical systems. One route to performing nanoNMR is magnetic resonance force microscopy, already used to image small organisms with nanometre resolution^2^. However, typical operating conditions of magnetic resonance force microscopy require ultralow temperatures and high vacuum^2^,^3^, which, unfortunately, are incompatible with most molecular processes of interest.

An alternate approach to NMR at the nanoscale makes use of individually addressable paramagnetic centres near the surface of a solid-state host to probe sample spin species in its immediate vicinity. Perhaps the most prominent example is the nitrogen-vacancy (NV) centre, a spin-1 defect in the diamond lattice formed by a substitutional nitrogen atom and an adjacent vacancy^4^. Recently, single NV centres separated only a few nanometres from the diamond surface were used to detect the NMR signal associated with the random magnetic spin noise of a nanoscale proton ensemble under ambient conditions^5^,^6^. Subsequent studies extended this initial work to other spin species and demonstrated improved detection sensitivity, attaining the limit of a few nuclear spins^7^,^8^,^9^,^10^,^11^. Furthermore, by articulating NV magnetometry with scanning microscopy, it has been possible to image the spatial distribution of nuclear spins within a polymeric phantom with about 10 nm resolution^12^,^13^.

In this study, we use shallow NVs to probe mesoscale proton ensembles from different organic substances deposited on the diamond surface. We resort to a form of correlation spectroscopy and reconstruct the equivalent of a nuclear free induction decay (FID), which, unlike the NMR counterpart, does not require nuclear spin pre-polarization. This pseudo FID—below referred to as correlation signal—has a limited decay time governed by the NV spin-lattice relaxation time *T*_1_ (typically longer than the NV coherence lifetime *T*_2_), which allows us to attain spectral resolution superior to that possible with standard magnetometry techniques. On applying this scheme to solid- and liquid-state substances we find substantial differences in the correlation signal envelope, which we associate with the presumably dissimilar molecular dynamics governing these systems. In particular, we observe long-lived ^1^H signals from oil molecules, which we interpret in terms of an interplay between molecular tumbling and self-diffusion.

## Results

**Figure 1:**
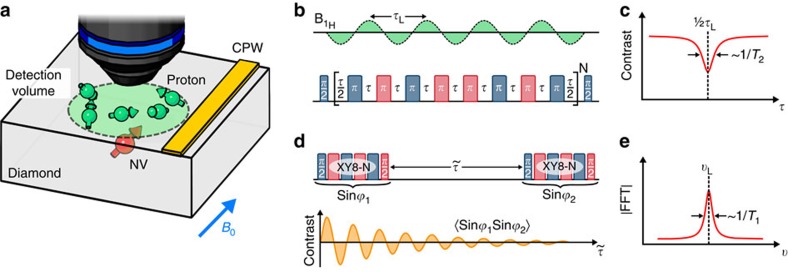
Schematics of the experimental set-up and basic detection protocol. (**a**) An organic sample is brought into contact with the diamond surface, and shallow NV centres are used as NMR detectors. (**b**) XY8-N multi-pulse sequence. A change in the NV response is observed when the interpulse separation *τ* matches half the Larmor period. The blue/red hue indicates different microwave (MW) phases, which are shifted 90° relative to each other. (**c**) Repeating the XY8-N sequence for multiple pulse spacings *τ* yields an effective CW spectrum of the nuclear spin noise. (**d**) Nuclear spin correlation protocol. Selective detection of the proton spins is attained by choosing the pulse spacing *τ* equal to half the Larmor period. The correlation signal shows a decaying oscillating behaviour as a function of the ^1^H spin evolution interval 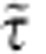. (**e**) The Fourier transform of the time domain signal yields the sample NMR spectrum. CPW, coplanar waveguide; FFT, fast Fourier transform.

Figure 1a depicts a typical NMR experiment using NV centres. The set-up consists of a home-built confocal microscope, which excites single NV centres in the illumination volume via a 532-nm laser. Green light initializes the NV—a spin-1 system—into the *m*_S_=0 level of its ground state triplet, which features a zero-field splitting of 2.87 GHz (ref. 14). The spin-state-dependent back fluorescence of the NV centre is collected via the imaging objective and is focused onto a single-photon detector. A moveable electromagnet provides a static magnetic field of about ∼25 mT and a nearby coplanar waveguide is used for the application of microwave irradiation at the frequency of the *m*_S_=0 ↔ *m*_S_=−1 transition (∼2.2 GHz). For the experiments herein we use single NV centres produced via 2.5 keV ^15^N^+^ ion implantation into a type IIa [100] diamond crystal (see Methods). The dipolar coupling to nuclear spins external to the diamond lattice is strong enough to imprint the NV response with a signature that originates from the statistical nuclear spin polarization^5^,^6^. The detection volume is roughly defined by the NV distance to the surface—about 5 nm in the present case—which approximately corresponds to ∼10^3^ protons for typical organic samples^5^,^6^,^10^.

A common way to detect the NMR signal via the NV centre is based on a quantum lock-in algorithm^15^,^16^, which is implemented through an XY8-N dynamical decoupling sequence^6^,^7^,^8^,^9^,^10^,^11^,^12^. Here a train of equidistant *π* pulses is used to selectively enhance the NV detection sensitivity at a frequency determined by the inverse pulse spacing *τ*^−1^. As shown in Fig. 1b, the XY8-N sequence is embedded within a Ramsey protocol (comprising two *π*/2 pulses), so as to convert the integrated effect of the nuclear spins—in the form of an accumulated NV phase shift—into a change in the NV fluorescence. The result of such a measurement is an effective continuous wave (CW) spectrum of the sample spins (Fig. 1c), whose linewidth is ultimately limited by the XY8-N coherence time 
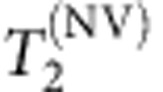
.

**Figure 2:**
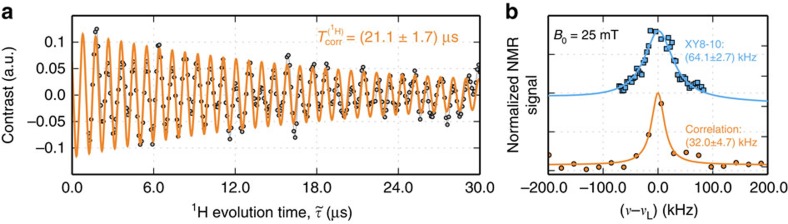
Time-resolved NMR of near-surface ^1^H spins. (**a**) XY8-3 correlation signal (circles) from protons in a solid polymeric mixture (sample A) in contact with the diamond surface. The yellow trace corresponds to an exponentially damped sinusoid with time constant of 21 μs and frequency equal to ∼1 MHz. The vertical axis indicates the contrast in a scale relative to the NV Rabi amplitude. (**b**) Comparison between the ^1^H-NMR spectra obtained with an XY8-10 sequence (blue squares) and after Fourier transformation of the XY8-3 correlation protocol shown in **a** (yellow circles). The blue and yellow traces, respectively, indicate Lorentzian fits to each data set, accompanied by their corresponding full-width at half-maximum values. Both curves are vertically displaced for clarity.

The coherence lifetimes of shallow NVs are often shorter than the characteristic timescales governing nuclear spins, thus complicating our ability to gather detailed spectroscopic information on the structure, chemical composition or dynamics of the system under investigation. One way to circumvent this limitation is the nuclear spin detection protocol of Fig. 1d^17^ designed to exploit the typically longer NV spin-lattice relaxation times 
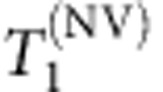
. The pulse sequence comprises two XY8-N trains separated by a variable interval 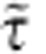; in each of them the interpulse separation is kept in sync with the sample spin Larmor precession, that is, we choose 
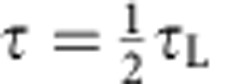
. During the evolution time 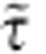 the magnetization information is stored in the longitudinal NV spin component while the sample nuclear spins are allowed to evolve. The underlying idea is that if the nuclear spin coherence loss is sufficiently slow, the phases *ϕ*_1_, *ϕ*_2_ picked up by the NV during the two consecutive XY8-N interrogations are correlated with each other. The latter results in a signal similar to an FID in conventional NMR, which, however, does not require nuclear spin (pre-)polarization. Interestingly, nuclear spin coherences lasting up to hundreds of microseconds—the typical 
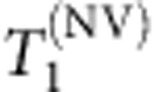
 time of the NVs we use here—can be probed with this technique, thus allowing us to better discriminate between different sample dynamics. In this light, our technique can be considered an alternative to double resonance schemes already used to reconstruct FID-like signals from ^1^H spins near shallow NVs^5^ while circumventing ambiguities on the nuclear spin species giving rise to the observed response^18^. Figure 2a shows the NV response in the presence of a solid organic film as a function of the evolution time 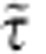. This system—a complex mixture of long-chain polymers hereby referred to as sample A—is formed by the adhesive used to affix the diamond crystal to the sample holder (Merckoglas). Similar to a conventional FID the correlation signal oscillates over tens of microseconds to gradually decay to zero. We find that the decay is reasonably described by an exponential, and has a characteristic time constant 
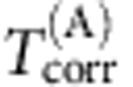
∼20 μs. After a cosine transformation, we find a peak centred at the ^1^H Larmor frequency (∼1.06 MHz) exhibiting a Lorentzian linewidth of ∼30 kHz, in agreement with that expected for static protons in a typical solid-state organic system. Of note, the correlation signal amplitude is only a small fraction (∼10%) of the maximum possible fluorescence contrast (30% between spin states *m*_S_=0 and *m*_S_=±1) (refs 14, 17). On the other hand, the resulting spectral linewidth is about a factor 2 smaller than that obtained with an XY8-10 sequence, which highlights the limitations inherent to sensing protocols governed by the coherence lifetime of the probe NV (Fig. 2b).

**Figure 3:**
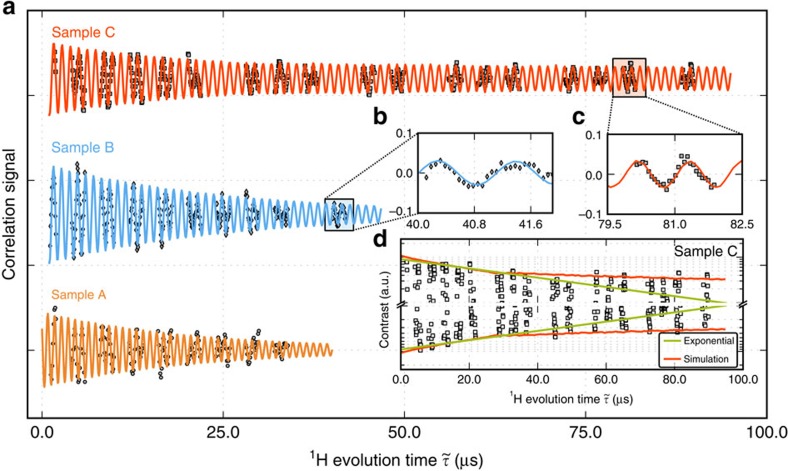
XY8-3 correlation signals for three different organic samples. (**a**–**c**) The data corresponding to both solid samples (samples A and B, respectively, orange and blue traces) match an exponentially damped sinusoid with time constants of ∼21 and ∼29 μs. By contrast, the correlation signal from protons in sample C exhibits a long-lived tail that outlives the exponential decay at early times (green trace in the (**d**)). The overall response can be reproduced semi-quantitatively via a model comprising a 1.5-nm layer of adsorbed molecules rotating about fixed positions and an outer section of self-diffusing fluid. Best agreement with the experimental observations is attained assuming translational and rotational diffusion constants of 0.3 nm^2^ μs^−1^ and 0.05 rad^2^ μs^−1^ for the outer and inner layers, respectively (red sinusoid trace in **a** and red envelope in **d**). Given the relatively low fluorescence contrast, only portions of the signal were measured. Though obtained with different NVs, each curve must be considered representative of the NV response for the corresponding proton ensemble under study.

Since the timescale probed in Fig. 2a is still considerably shorter than 
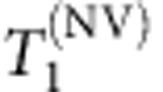
, a natural question is whether this technique can be exploited to investigate longer-lived nuclear spin coherences arising, for example, from alternate forms of motional narrowing. A first step in this direction is shown in Fig. 3 where we compare representative correlation signals from different organic systems, including that in Fig. 2 as well as a softer polymeric film (polydimethylsiloxane (PDMS)) and a drop of immersion oil (Fluka Analytical, 10976) in direct contact with the diamond surface (see Methods and Supplementary Note 1). Below we refer to the latter two systems as samples B and C, respectively. In all three cases we observe periodic oscillations reflecting the already highlighted precession of proton spins about the applied magnetic field. However, the time over which these oscillations last and, perhaps more importantly, the way the signal envelope changes over time differ significantly in each case. In particular we find that the correlation signal from sample C exhibits a long-lasting tail extending to about 80 μs (possibly limited by NV spin-lattice relaxation, see Fig. 3c). Unlike sample A, this behaviour cannot be captured by a single-exponential envelope (Fig. 3d) and thus points to differing nuclear spin relaxation mechanisms. Sample B, on the other hand, shows a somewhat intermediate, longer-lived signal, which, nonetheless, does not depart from the single-exponential response observed in sample A (Supplementary Figs 1 through 3).

## Discussion

To gain a more quantitative understanding of the mechanisms at play, we start by modelling the NV response in a way that accommodates the different dynamics governing solid and liquid samples. Using a semi-classical approximation to describe the NV interaction with the nuclear spin bath (Supplementary Notes 2 and 3 as well as Supplementary Figs 4 and 5) and assuming all molecules move independently, we write the correlation signal as


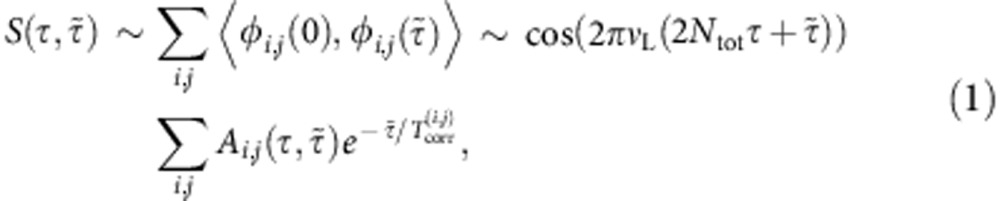


where the sum extends over all proton spins *i* in the *j*-th molecule and *ϕ*_*i*,*j*_ denotes the corresponding contribution to the accumulated NV phase, *A*_*i*,*j*_ is the resulting signal amplitude, *ν*_L_ is the nuclear Larmor frequency and *N*_tot_ is the total number of *π* pulses in each XY8-N train. The signal amplitude *A*_*i*,*j*_ is proportional to the root mean square field generated by the proton spins at the position of the NV centre and varies with different molecule positions and/or orientations over the evolution time 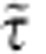. In the case of a solid, molecules occupy fixed positions and nuclear spin relaxation is dominated by the nuclear dipolar interactions, presumably homogeneous throughout the sample. In this limit, the rate 
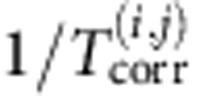
 describing the spin relaxation of nuclear moment *μ*_*i,j*_ approaches a uniform value, and equation (1) converges to an exponentially damped sinusoid (Supplementary Note 2), the case observed for both solid samples. For sample A, we find 
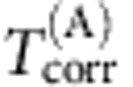
∼21 μs, which corresponds to a decay dominated by static dipolar couplings, of order *μ*^2^/|Δ*r*|^3^∼30 kHz for typical inter-proton distances |Δ*r*|∼0.1 nm in organic samples. The slightly longer coherence lifetime in sample B, 
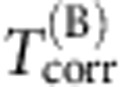
∼29 μs, possibly originates from an enhanced molecular mobility in this material^19^,^20^.

Fluidic systems, on the other hand, differ from solids in that molecules experience a markedly distinct dynamics dominated by fast tumbling and self-diffusion. Both mechanisms contribute to average out the internuclear spin couplings and thus lead to longer nuclear spin coherence lifetimes. However, given the nanoscale detection volume of a shallow NV—roughly restricted to a half-sphere of diameter equal to the NV depth^6^,^21^—molecules interacting with the probe paramagnetic centre may exchange with the bulk of the system at some arbitrary time during the protocol. This situation is somewhat reminiscent of that found in fluorescence correlation spectroscopy, where molecules diffuse in and out of the detection volume^22^,^23^,^24^. In the present case, we simplify the problem by assuming that molecules occupy frozen positions during each interrogation interval, thus instantaneously tagging the NV with a phase shift corresponding to the molecule positions at times 0 and 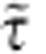. In this limit, we rewrite the NV response as


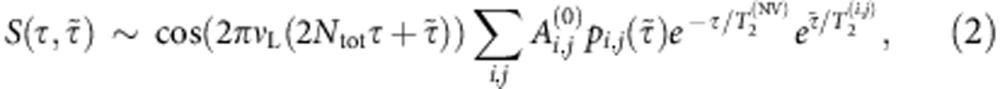


where 
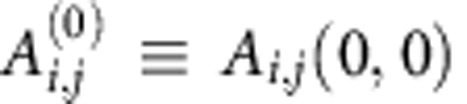
, 
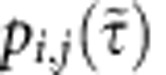
 denotes the conditional probability of nuclear spin (*i*,*j*) remaining within the detection volume over the correlation interval 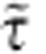 and 
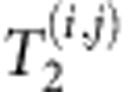
 is the nuclear spin transverse relaxation time. The correlation decay of equation (1) 
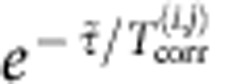
 is expressed as the product of the NV decay 
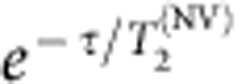
, the conditional probability 
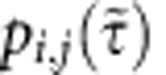
, and the NMR decay of the sample spins 
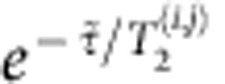
. For simplicity, we assumed in equation (2) that the signal amplitude *A*_*i,j*_, and 
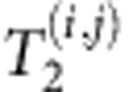
 remain unchanged after self-diffusion during 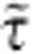, which is consistent with a bimodal distribution where molecules either self-diffuse or not depending on their location at time zero.

**Figure 4:**
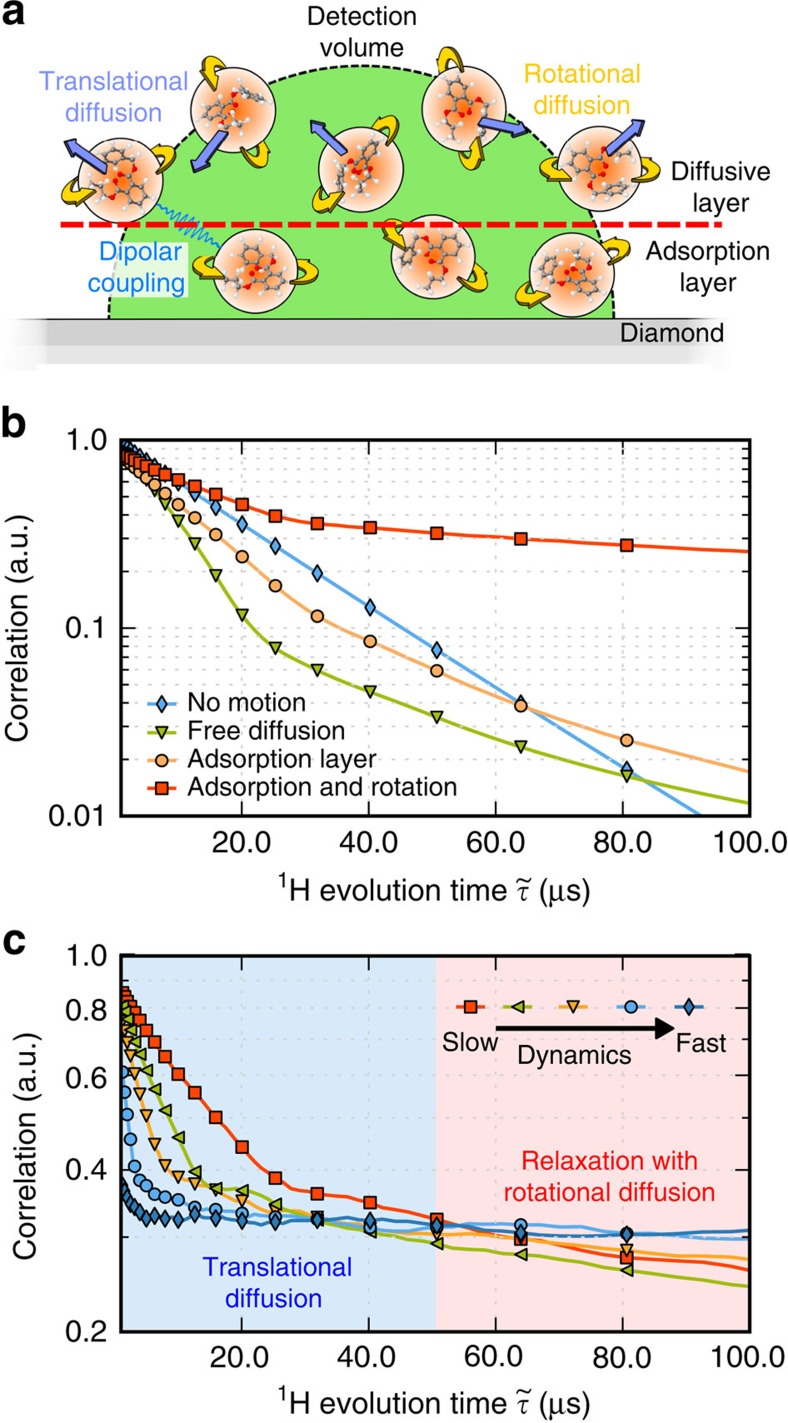
Dynamics of the fluid near the solid–liquid interface. (**a**) We assume a layered model in the detection volume (green semi-sphere) comprising a static adsorption film (red dashed line) where translational diffusion (light blue arrows, rotational diffusion as yellow arrows) of the molecules (orange spheres) is prohibited, and a mobile, outer section in which molecules self-diffuse as in a bulk liquid. (**b**) Simulated correlation signal for varying sample conditions. For the case of a rigid polymer the signal decay is dominated by internuclear dipolar coupling (blue trace). In the opposite limit of an unrestricted bulk liquid, the signal amplitude quickly decreases due to molecules leaving the detection volume during the inter-sequence time (green trace). Combining the two regimes via an adsorption layer separating the diamond surface from the bulk liquid yields an intermediate response (orange trace). Our experimental observations are best described by allowing molecules in this layer to rotate about their equilibrium positions (red trace). The translational self-diffusion coefficient used for the bulk fluid is 0.3 nm^2^ μs^−1^ (green, yellow and red traces); the rotational diffusion coefficient in the adsorbed layer is 0.05 rad^2^ μs^−1^ (red trace). (**c**) For a fixed adsorbed layer thickness (1.5 nm), we increase the translational self-diffusion coefficient in the outer section of the fluid and the rotational diffusion constant on the surface from 0.3 nm^2^ μs^−1^–0.05 rad^2^ μs^−1^ (red trace) to 0.6 nm^2^ μs^−1^–0.05 rad^2^ μs^−1^ (green trace), 1.0 nm^2^ μs^−1^–0.10 rad^2^ μs^−1^ (yellow trace), 2.5 nm^2^ μs^−1^–0.25 rad^2^ μs^−1^ (light blue trace) and 10 nm^2^ μs^−1^–1.00 rad^2^ μs^−1^ (dark blue trace). Note the two sections of the graph, dominated either by molecular diffusion or rotation.

Although the dynamics of molecules at a solid–liquid interface are not fully understood, several studies suggest that solids induce order in adjacent fluids^25^. The boundary condition typically invoked is one where the liquid is (nearly) static over the surface^26^. In particular, recent atomic force microscopy experiments in water indicate that the transition to bulk fluid dynamics is abrupt and takes place on the nm range, depending on the surface hydrophobicity^27^. To numerically calculate 
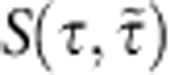
 in equation (2) we divide the detection volume in two layers (Fig. 4a): molecules adjacent to the surface rotate about fixed positions, while molecules in the outer layer rotate and physically diffuse away from the NV. The set of conditional probabilities 
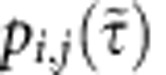
 for the mobile layer is calculated from a Monte Carlo run assuming a bulk diffusion coefficient for the fluid. To bring the number of free parameters to a minimum, we tie the rotation rate of bulk molecules to the diffusion constant via the Stokes–Einstein and Debye–Stokes–Einstein equations; comparison between the observed and calculated signals is carried out by systematically varying the rigid layer thickness and self-diffusion constant of the fluid (Supplementary Notes 3 and 4).

To illustrate the impact of the dynamics of molecular diffusion and rotation on the correlation signal, we use equation (2) to calculate the envelope governing the decay of 
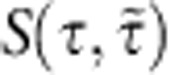
 assuming different boundary conditions. For example, the green trace in Fig. 4b shows the response anticipated in the case where the sample dynamics corresponds to that of a bulk fluid up to the very surface of the diamond crystal, that is, we assume the layer of adsorbed molecules has negligible thickness. Using a diffusion constant *D*_oil_∼0.3 × 10^−12^ m^2^ s^−1^ (comparable to that anticipated for this sample) we find that the calculated signal envelope somewhat reproduces the trend in our experiment, namely, it exhibits a long-lasting tail that outlives the exponential decay at early times (green trace in Fig. 4b). A numerical analysis shows that this tail stems from the longer nuclear spin coherences inherent to the mobile molecules of a fluid. Quantitatively, however, the calculated envelope does not agree well with the experimental data: First we note that the relative contribution of the long-lasting signal is comparatively small (because most molecules leave the detection volume when 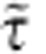 is sufficiently long). Further, compared with the case where no motion is present (infinite rigid layer, blue trace in Fig. 4b), we find a much faster initial decay (also absent in the experimental signal of Fig. 3). Changing the diffusion constant to greater or smaller values either accelerates the initial decay or further reduces the tail, thus pointing to the inadequacy of the underlying model (Supplementary Note 4).

We can, however, reproduce the experimental data when we assume the presence of a 1.5-nm-thick adsorption layer (orange trace in Fig. 4b), particularly if molecules within this layer are allowed to rotate about their equilibrium positions (red trace in Fig. 4b). The observed and calculated correlation signals for the fluidic sample are superimposed in Fig. 3b (red trace, compare also Supplementary Figs 6 and 7). The result captures reasonably well both the initial decay rate and the amplitude of the long-lived contribution. From the transverse relaxation rate 
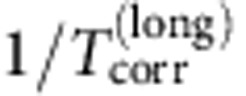
≈3 kHz assigned to adsorbed protons we determine the rotational correlation time 
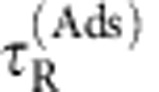
≈3.3 μs, much longer than in the outer segment of the sample (
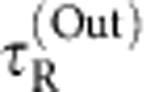
∼190 ns as calculated from the Stokes–Einstein equation for *D*_oil_∼0.3 × 10^−12^ m^2^ s^−1^ (ref. 22)). We caution, however, that this value must be interpreted as an upper limit given the likely impact of NV spin-lattice relaxation on the correlation signal of sample C (see Supplementary Notes 4 and 5 as well as Supplementary Figs 8 and 9).

In the light of the above discussion, it is important to consider the time window the present technique is sensitive to. For example, Fig. 4c shows that doubling the diffusion constant from 0.3 × 10^−12^ to 0.6 × 10^−12^ m^2^ s^−1^ leads to a significant change of the predicted signal envelope, which more quickly converges to the longer-lived tail produced by the adsorbed nuclei. In particular, molecules diffusing distances >10 nm on a microsecond scale—corresponding to self-diffusion coefficients of order ∼10^−11^ m^2^ s^−1^ or greater—leave a negligible imprint on the correlation signal. Correspondingly, fluidic systems such as water (*D*_water_*∼*2.3 × 10^−9^ m^2^ s^−1^) would be detectable only through the formation of a semi-mobile layer adjacent to the diamond surface. We emphasize that the sensitivity to the local system dynamics is not immediately indicative of accuracy in the derived parameters, which here must be understood as moderate given the crude assumptions of our model (spherical versus linear molecules, isotropic versus anisotropic diffusion, etc., see Supplementary Note 4 and Supplementary Fig. 8). Along the same lines, we mention that it is difficult to ascertain whether the thin adsorbed layer observed in our experiments strictly originates from molecules in the fluid. In particular, recent studies have shown the presence of a protonated layer of comparable thickness possibly formed by water molecules or other hydrocarbons adsorbed on ambient exposure^12^,^13^. Controlled preparation of the diamond surface combined with NV-based NMR of spin species other than protons (for example, ^19^F or ^31^P) can provide the means to more precisely separate contributions from sample molecules structured near the solid–liquid interface. Finally, deeper NVs (for example, 15–20 nm from the surface) could be used to increase the relative contribution from more distant nuclear spins not adsorbed on the diamond surface, though at the expense of lower detection sensitivity and spatial resolution as well as longer interrogation times.

On a final note, we hypothesize that the impact of the diamond crystal on the near-surface dynamics of the organic system is not restricted to fluidic samples but more in general, extends to most soft condensed matter systems. In particular, comparison between NV-detected and inductively detected NMR signals—omitted here for brevity—reveals longer coherence lifetimes for nuclear spins within the bulk of solid samples A and B (200 and ∼500 μs, respectively), which we interpret as indicative of a more restricted dynamics near the diamond crystal. Naturally, this conclusion relies on the connection between the nuclear spin coherence lifetime and the molecular mobility in these systems, a notion we confirm by examining control samples engineered to exhibit a variable degree of rigidity (Supplementary Note 5 and Supplementary Figs 10 and 11).

In summary, the results herein introduce a new strategy to nanoscale nuclear spin sensing where the signal is recorded in the form of an FID without the need for nuclear spin polarization or molecular labelling. The inherently small detection volume—of order 100 nm^3^ in the present case—makes this form of sensing ideal to investigate the dynamical processes on mesoscales, hard to access with other experimental techniques. For example, our approach could be exploited to more clearly expose the role of nanoscale surface roughness on the dynamics of flow, or to experimentally test differing boundary conditions invoked at the liquid–solid interface^25^,^26^ (for example, no slip, multilayer locking, etc). Extensions articulating the present technique with known NMR protocols (for example, homo- or heterospin decoupling sequences^28^) can be used to shed light on the chemical composition of adsorbed films in cases where the dynamics are insufficient to suppress internuclear couplings. In particular, nuclear spin manipulation in the form of radio-frequency multipulse sequences can be applied during 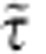 without deleterious effects on the NV response. Likewise, spin swap schemes—designed to exchange the spin states of the NV and its ^14^N (or ^15^N) host during the correlation interval^17^—provide a route to probe slow dynamical processes on timescales exceeding the NV longitudinal relaxation time^6^. Studies in this regime—more susceptible to the limited fluorescence contrast affecting the present correlation protocol—can benefit from the enhanced photon collection efficiency recently demonstrated for NVs within engineered diamond nanostructures^29^,^30^,^31^.

More in general, the present technique opens interesting opportunities for the investigation of chemical or biochemical systems affected by compositional heterogeneities or local aggregation. Cell membranes in particular could serve as a fascinating research platform, since molecular diffusion is non-Brownian and mostly restricted to nanoscale fluidic pockets (presumably) separating more rigid structures^32^. By the same token, experiments as a function of temperature and/or the composition of the bulk fluid can help explore various thermally or chemically activated processes including, for example, protein folding or the dynamics of molecular motors tethered to the diamond surface.

## Methods

### Diamond samples

We use Type IIa CVD grown (100)-oriented diamond samples from Element6. The NV centres used in this study were generated via 2.5 keV ^15^N^+^ ion implantation into the diamond substrates. The substrates were subsequently annealed at 240 °C for 2 h, followed by an 8-h annealing at 850 °C temperature, both in high vacuum. Afterwards the diamonds were boiled in a 1:1:1 mixture of H_2_SO_4_, HClO_4_ and HNO_3_, to remove any residual graphitic contaminations from the diamond surfaces.

### Sample preparation

We measured the correlation signals for three different organic samples, two organic polymers (A and B) and a liquid sample (C).

For the measurements of the solid compounds we spin coated a visibly thick layer of the respective material onto the diamond surface. Sample A is a ‘liquid coverslide' material dissolved in toluene (Merckoglas, Merck), which has a refractive index similar to glass and immersion oil. We dilute the Merckoglas base solution with toluene in a 1:1 ratio before spin coating. For the analysis we treat Merckoglas as a typical organic polymer, such as poly(methyl methacrylate), and assume a comparable proton density.

Sample B is a film of PDMS. For this purpose a droplet of PDMS (Sylgard 184 Silicone Elastomer, Dow Corning in a 10:1 mixing ratio of the base to the curing agent) was spin coated onto the diamond surface. Afterwards the PDMS is annealed by placing the diamond onto a hotplate for 2 h at 80 °C. We note that the signal-to-noise ratio under the PDMS coating is much smaller than for the other two samples, and requires longer measurement times. This is due to the larger mismatch of refractive indices, and the accompanied loss of fluorescence signal.

The measurements of the liquid sample C were performed by covering the diamond surface with immersion oil (Fluka Analytical, 10976).

Before/after the measurements, the samples were removed by rinsing the diamond in a solvent while sonicating. The diamond is subsequently boiled in the above-mentioned 1:1:1 acid mixture for multiple hours to remove any organic residues of the sample or solvent from the surface.

To ensure the observed NMR signal (mainly) originates from the sample rather than from adsorbates due to exposure to the ambient environment, the diamond is removed from the acid and immediately covered with the respective sample, so as to minimize the exposure time to the ambient.

## Additional information

**How to cite this article:** Staudacher, T. *et al*. Probing molecular dynamics at the nanoscale via an individual paramagnetic centre. *Nat. Commun.* 6:8527 doi: 10.1038/ncomms9527 (2015).

## Supplementary Material

Supplementary Information Supplementary Figures 1-11, Supplementary Notes 1-5 and Supplementary References
