# Supplementary material for: Probing molecular dynamics at the nanoscale via an individual paramagnetic centre
Source: Nat Commun. 2015 Oct 12;6:8527. doi: 10.1038/ncomms9527 (PMC4633823; doi:10.1038/ncomms9527)
Supplement: Supplementary Information — Supplementary Figures 1-11, Supplementary Notes 1-5 and Supplementary References [file ncomms9527-s1.pdf]

SUPPLEMENTARY FIGURES

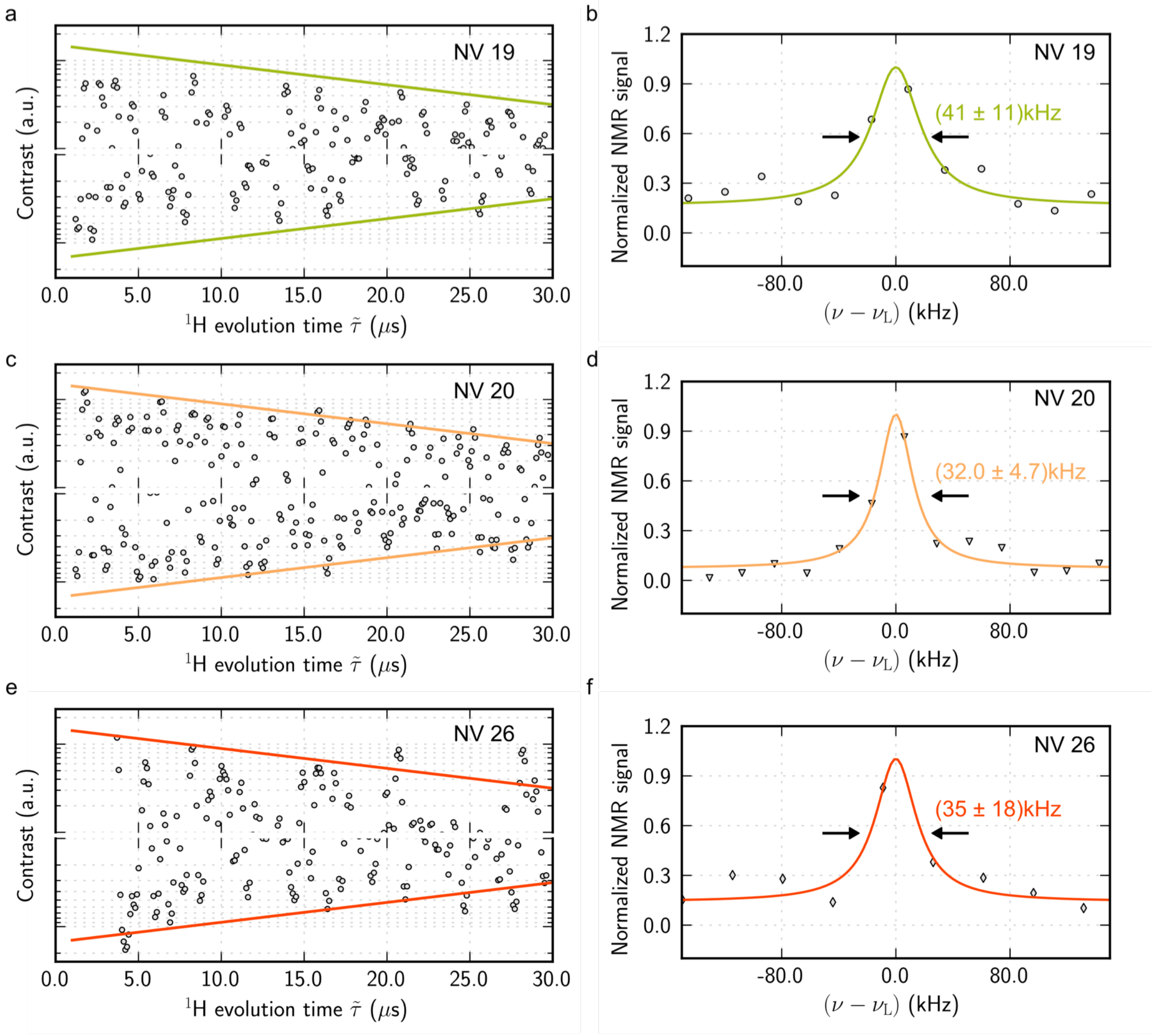

Supplementary Figure 1. Signal reproducibility for Sample A. (a) Proton correlation signal as detected from an NV center referred to as NV 19 (open circles) for Sample A (Merckoglas). The solid trace is the envelope of our model adapted to a fully static proton bath. (b) Fourier transform (magnitude mode) of the signal in (a) centered at the proton Larmor frequency ν_L along with a Lorentzian fit (solid green trace). (c,d) Same as in (a,b) for NV 20. (e,f) Same as in (c,d) but for NV 26.

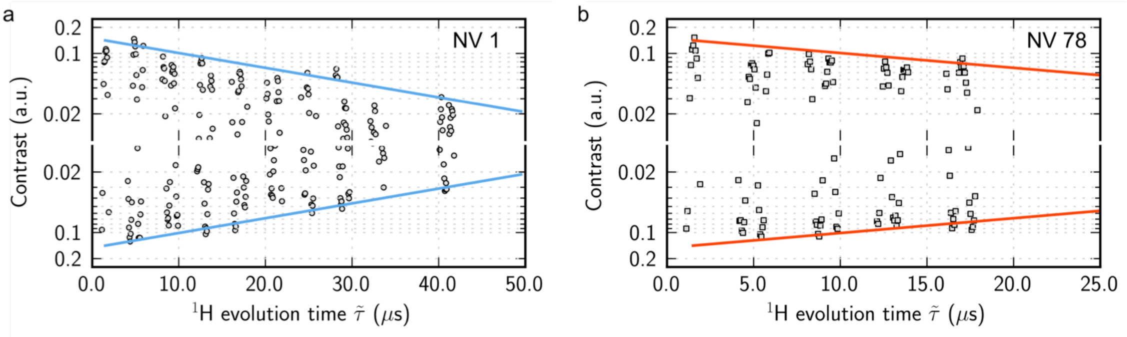

Supplementary Figure 2. XY8-3 correlation signal under a coating of sample B for two different NV centers. The time domain data is compared with a decay envelope calculated assuming partial motional narrowing due to slow molecular dynamics as described above.

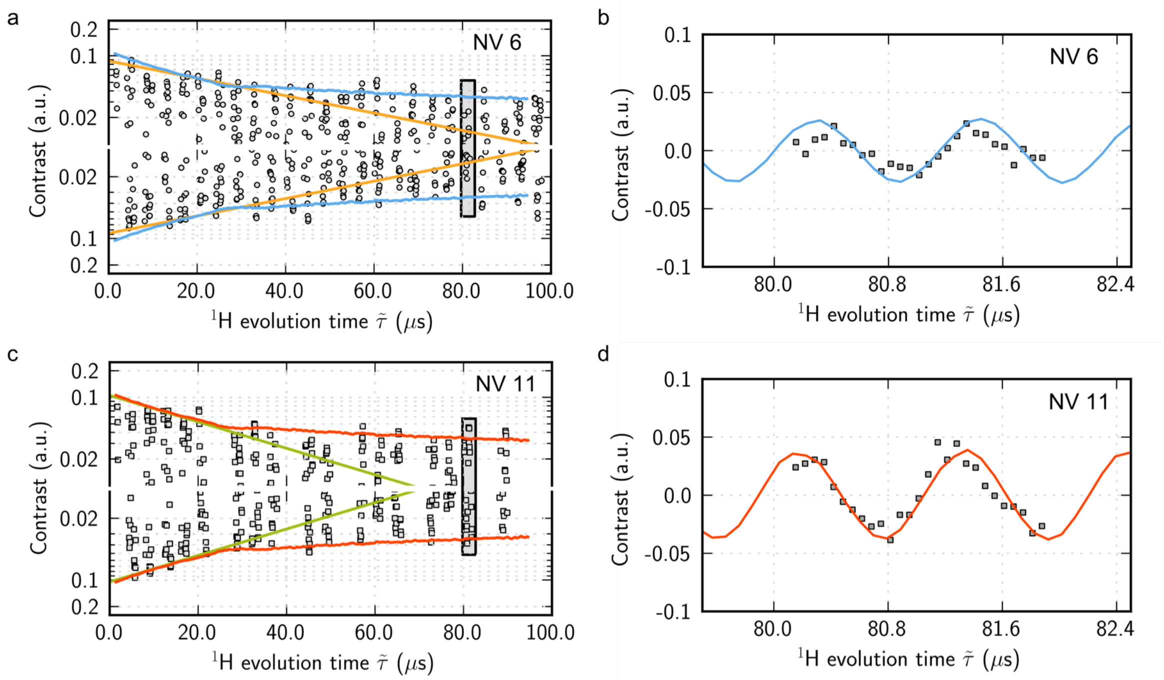

Supplementary Figure 3. Signal reproducibility in the presence of a liquid. (a) Proton correlation signal from a shallow NV (referred to as NV 6, open circles) in the presence of immersion oil. The yellow and light blue traces are the envelopes of an exponentially decaying sinusoidal and the presented model, respectively. The exponential fit considers the initial decay $\tilde{\tau} \leq 25$ μs whereas the model considers contributions from molecules in the bulk liquid and semi-mobile molecules from an adsorbed layer as described in the main text. (b) Zoomed image of the data points within the shaded area in (a); the light blue trace is the segment of a fit to a sinusoidal function at the proton Larmor frequency scaled by the envelope in (a). (c,d) Same as in (a,b) but for a different NV (identified as NV 11 in the image). In both cases we use the same parameters, namely, the adsorbed film thickness as well as the translational and rotational diffusion coefficients.

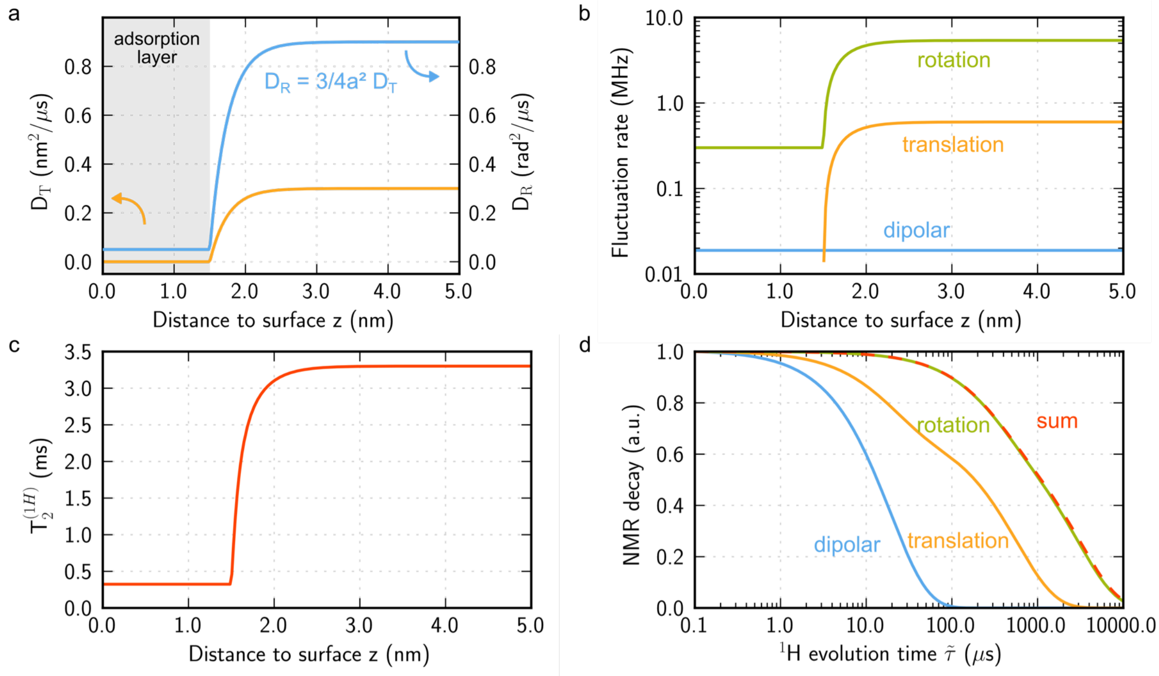

Supplementary Figure 4. Calculation of the nuclear spin relaxation process. (a) Translational and rotational diffusion constants with increasing distance to the diamond surface. The rotational constant has a finite value at the surface, and both constants are linked via the Stokes-Debye-Einstein relations in the outside layer. (b) The varying diffusion constants result in different fluctuation rates throughout the detection volume, the dipolar interaction is constant, as we assume a constant proton density throughout the volume. (c) The relaxation time is dominated by the strong dipolar interactions in the static adsorption layer but quickly prolongs with faster molecular dynamics. (d) Integrated NMR decay over the detection volume, for different molecular interactions. The NMR signal quickly decays in the absence of molecular dynamics (blue curve), gets longer in the presence of translational diffusion (yellow curve), and is maximum if also rotational diffusion is allowed (green, red curve). Same parameters as in the main manuscript: molecule radius of a = 0.5 nm, proton density $\boldsymbol{\rho}$ = 50 nm^{-3}, adsorption layer thickness z_{ads} = 1.5 nm, surface translational diffusion constant of $D_{T,surface}$ = 0.0 $nm^2/\mu s$, bulk translational diffusion constant of $D_{T,bulk}$ = 0.3 $nm^2/\mu s$, a rotational diffusion constant on the surface of $D_{R,surface}$ = 0.05 $rad^2/\mu s$, and a rotational diffusion constant in the bulk of $D_{R,bulk} = 3/4a^2\, D_{T,bulk}$.

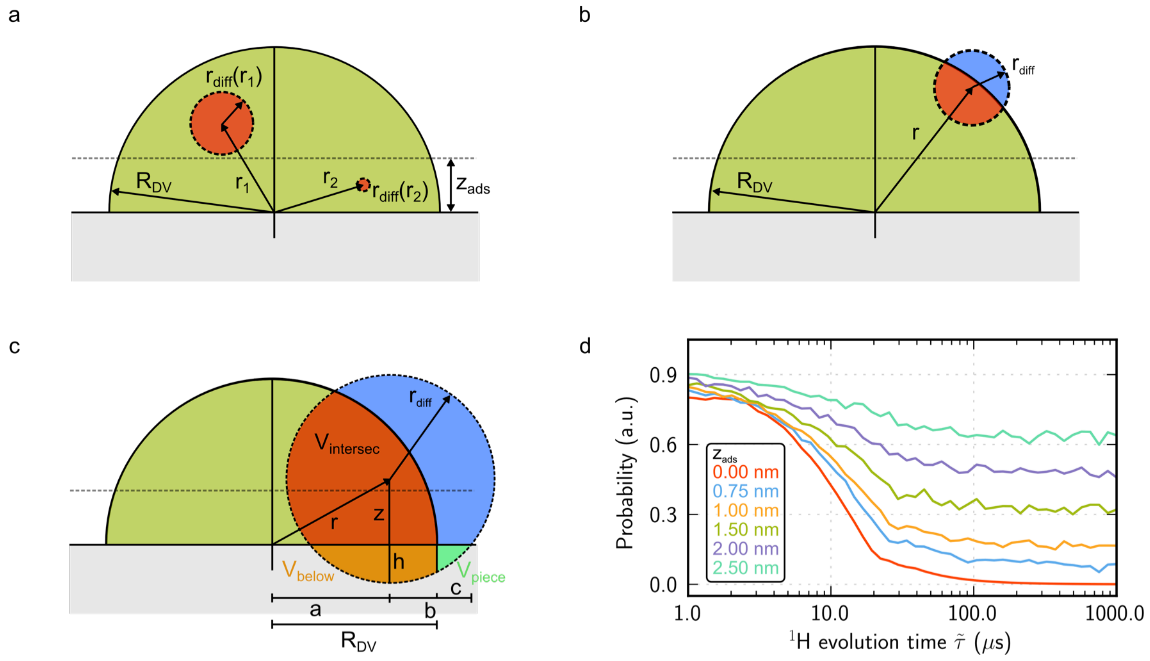

Supplementary Figure 5. Geometric model for signal loss due to translational diffusion (a-c) The detection volume (green) can be approximately described by a heterogeneous hemisphere comprising a static near-surface layer and a mobile outer segment. The probability of a molecule staying within the detection volume during $\tilde{\tau}$ depends on the assumed translational diffusion constant in the outer section of the hemisphere. This probability (growing from (a) to (c)) is described by the overlap (red) of the diffusion sphere with the detection volume. (d) For a translational diffusion constant of $D_{T,bulk}$ = 0.3 $nm^2/\mu s$, we find that the probability of molecules staying inside the detection volume quickly diminishes (red curve), unless a sizeable adsorption layer is present.

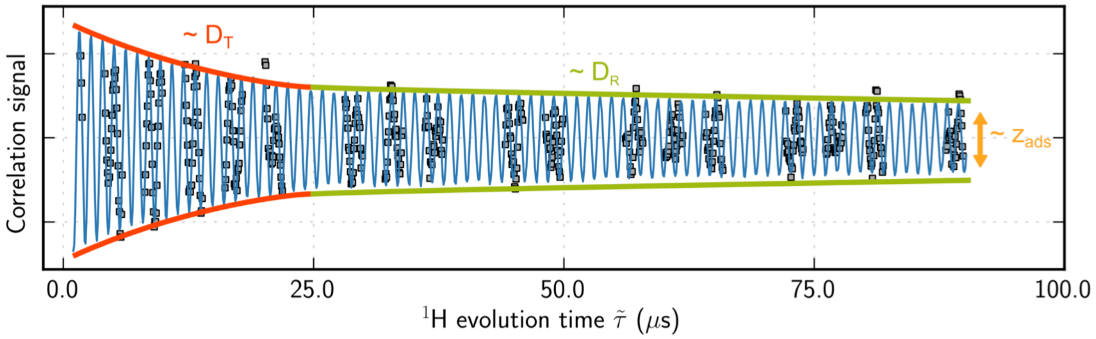

Supplementary Figure 6. Comparison of the MC simulation with the immersion oil data. A scaled simulation (red/green trace) is overlaid over the experimental data points (grey squares). The blue curve is a fit to a sinusoidal using the simulation as an envelope. The parameters used for the simulation were a molecule radius of a = 0.5 nm, proton density $\boldsymbol{\rho}$ = 50 nm^{-3}, adsorption layer thickness z_{ads} = 1.5 nm, bulk translational diffusion constant of $D_{T,bulk}$ = 0.3 $nm^2/\mu s$, and a rotational diffusion constant on the surface of $D_{R,surface}$ = 0.05 $rad^2/\mu s$. The red (green) segment of the envelope highlights the part of the decay dominated by translational (rotational) diffusion from molecules in the bulk fluid (adsorbed to the diamond surface). The amplitude of the green segment depends on the thickness z_{ds} assumed for the adsorbed layer.

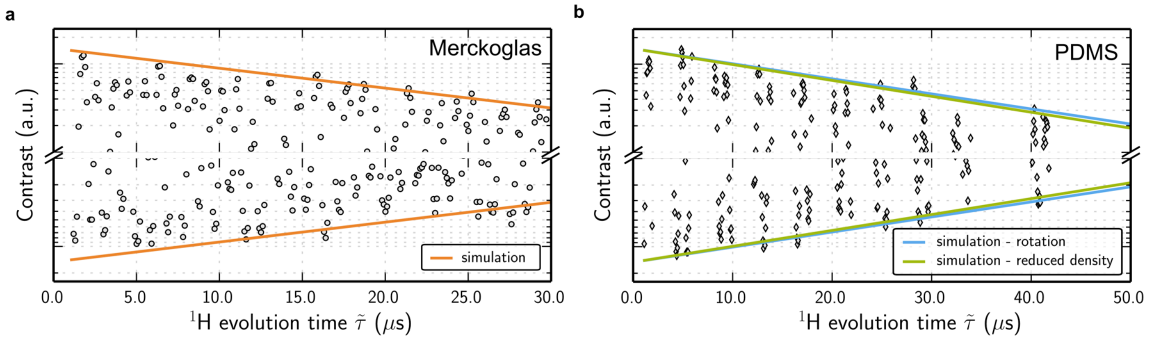

Supplementary Figure 7. Simulated correlation envelopes for the solid-state samples (a) The Merckoglas data can be readily described by a completely static bath of molecules, i.e. by a purely dipolar NMR relaxation, yielding a single exponential decay envelope (yellow curve), in good agreement with Eq. 1 in the main manuscript. (b) The PDMS data can be readily reproduced by either assuming slow molecular rotation with no center-of-mass translation (blue curve, $D_{R,surface}$ = 0.001rad^2/µs), or by assuming a completely static layer with a (~20%) reduced proton density of 40 nm^{-3} (green curve).

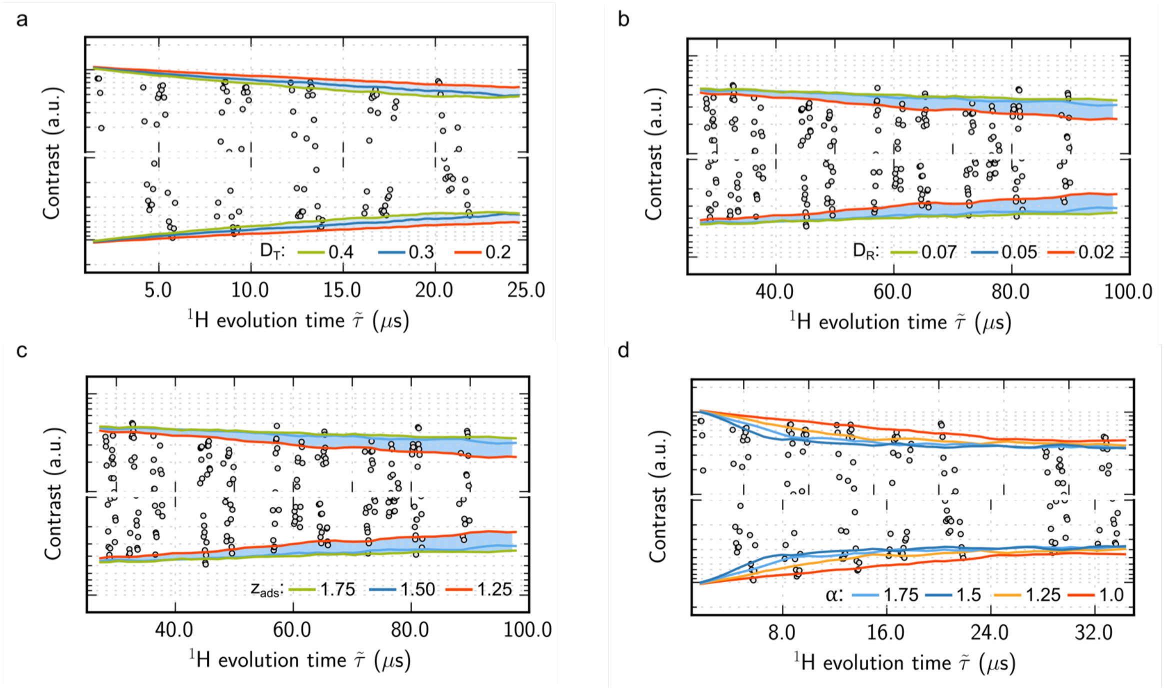

Supplementary Figure 8. Variation of the model parameters and influence in the obtained decay envelope. (a) Variation of the translational diffusion coefficient $D_{T,bulk}$, the respective value is noted in units of [$nm^2/\mu s$]. (b) Variation of the rotational diffusion coefficient in the semi-mobile adsorption layer $D_{R,surface}$, the respective value is noted in units of [$rad^2/\mu s$].. (c) Variation of the adsorption layer thickness z_{ads}, the respective value is noted in units of [nm]. (d) Influence of anomalous diffusion on the Initial signal decay. In (a) through (d) the data points correspond to NV 6 and the diamond coating is sample C (immersion oil). In all cases only the noted parameter is changed while the others have a fixed value coincident with that noted in the main text.

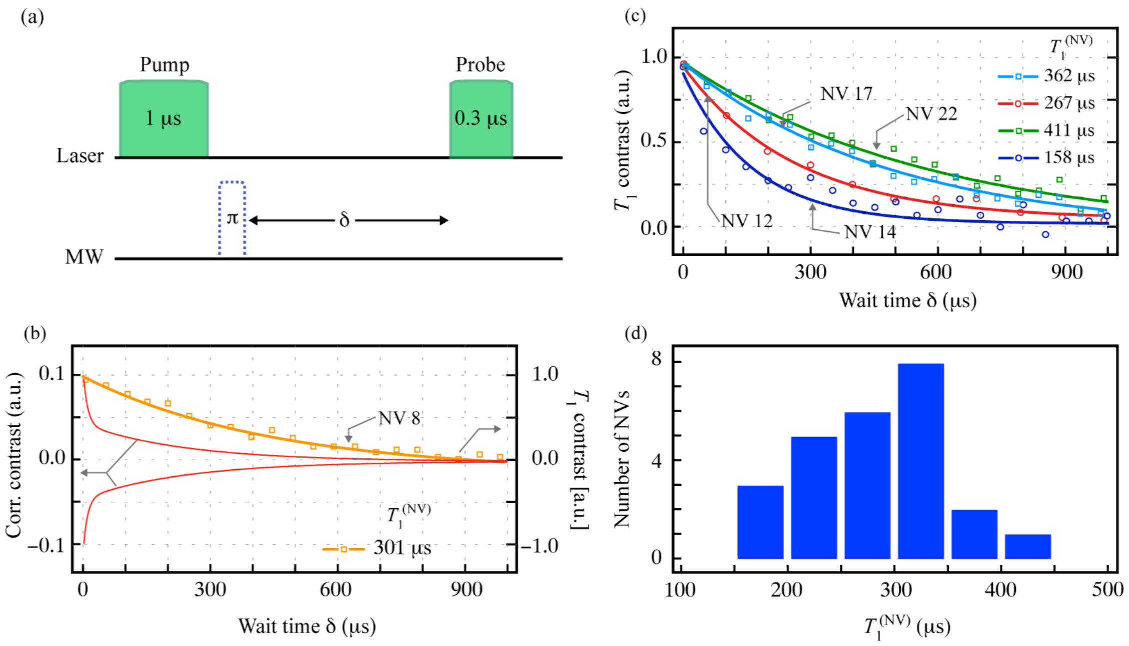

Supplementary Figure 9. Impact of NV spin-lattice relaxation on the correlation signal. (a) We determine $T_1^{(NV)}$ by subtracting the NV signals from pump-probe and inversion recovery protocols (both differ only on the application of a π-pulse immediately after the pump laser pulse). (b) Representative $T_1^{(NV)}$ measurement (squares); the solid line is a fit to an exponential decay with time constant $T_1^{(NV)} = 301$ μs. For comparison, the red trace reproduces the (extrapolated) envelope of the correlation signal for Sample C; the long-lived section of the curve decays with a characteristic time $T_{corr}^{(long)}$~250 μs. (c) Out of the 25 NVs investigated, most NVs spin-relax with a characteristic time of ~300 μs. The data sets in the plot are to be considered representative of the NV ensemble. (d) Histogram with the distribution of NV spin-lattice relaxation times.

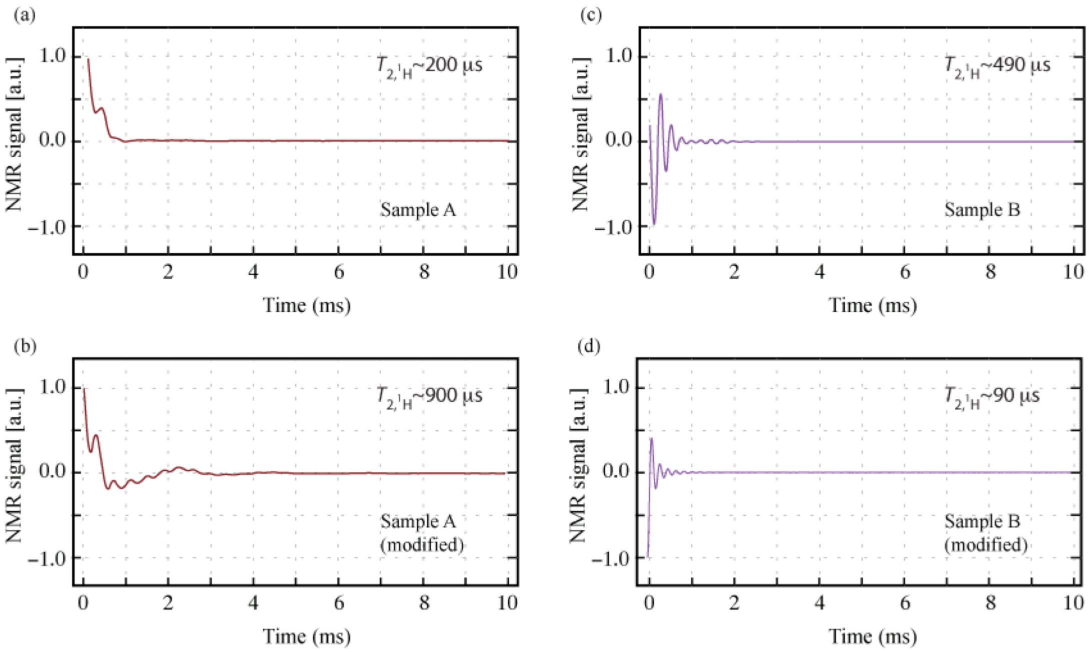

Supplementary Figure 10. High-field NMR of the solid samples. (a) ^{1}H NMR free induction decay from a dried 1:1 solution of Merckoglas in toluene (as used in our NV experiments). (b) Same as in (a) but for dried Merckoglas (without added toluene). (c) ^{1}H NMR signal from bulk PDMS; the elastomer/curing agent ratio is identical (10:1) to that used in our NV experiments. (d) Same as in (c) but for 'rigid' PDMS (6:1 mixing ratio). All NMR experiments were carried out at 14.1 T (600 MHz proton frequency) under ambient conditions.

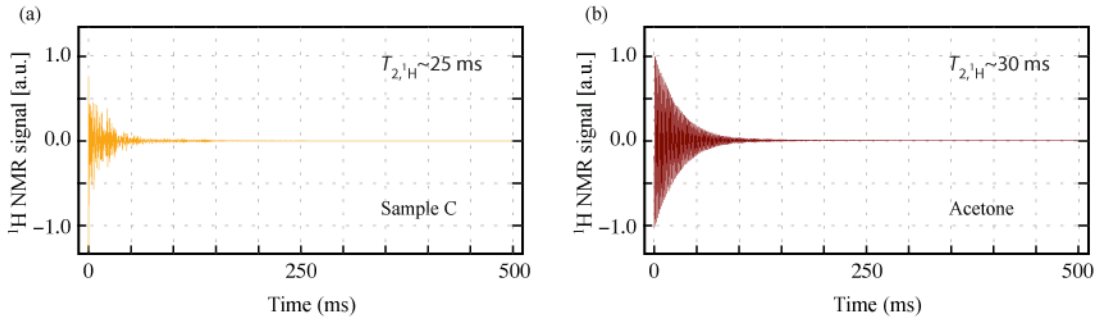

Supplementary Figure 11. ^{1}H NMR signal from Sample C. (a) ^{1}H free induction decay from a sample of the immersion oil used in our NV experiments. The proton spin transverse relaxation time is approximately 25 ms, limited by field inhomogeneity. (b) FID from protons in acetone. All experiments were carried out at 14.1 T (600 MHz proton frequency) under ambient conditions.

SUPPLEMENTARY NOTES

Supplementary Note 1

Signal reproducibility

All experiments were carried out with the same diamond sample, and thus with the same set of shallow NVs. However, because the diamond crystal must be physically removed from the microscope for surface coating, it is difficult to use the same individual NV for testing nuclear spins from different sample films. To circumvent this complication, we collected data from multiple (50 to 100) individual NVs exposed to the same sample film (see below). Among these only a few can be dynamically decoupled for a time sufficiently long to see the nuclear spin signature via an XY8-N sequence (a likely result of the NV depth dispersion and heterogeneity of the local concentration of paramagnetic impurities). We find that not every NV center exhibiting a nuclear-spin-induced dip under the XY8-N sequence also shows detectable nuclear spin correlation. The reason for this is still not fully understood but it may relate to the fortuitous overlap between the proton spin signature and the fourth harmonic of the ^{13}C-induced dip (see below): The latter can be easily mistaken by the former in an XY8-N sequence but not in the correlation protocol (where the nuclear spin Larmor frequency is directly probed). Also worth noting is the small relative amplitude of the correlation signal ($\approx 10\%$ of the maximum possible fluorescence contrast between the NV spin states), which leads to a correspondingly low SNR and makes detection difficult. Within these experimental limitations, Figs. 2 and 3 in the main text show *representative* data sets from the NVs that did display a sizeable correlation signal. Among the latter, excellent reproducibility was observed between data sets in all the samples we explored, as shown immediately below.

Sample A – Merckoglas

We examined a set of 52 NV centers, out of which 10 could be dynamically decoupled to coherence times of ≈ 100 μs, allowing us to detect the proton signal via an XY8-10 sequence. Only 4 of these NVs show a detectable signal after an XY8-3 correlation sequence.

Supplementary Figure 1 shows three of those NV centers, the one presented in the main text (NV20) and two additional ones. We find a reproducible correlation signal, described by an exponentially damped sinusoid centered around the proton Larmor frequency. The decay envelope (here maintained unchanged for all NVs in the figure) is qualitatively captured by the presented model, with a decay time of 20 μs.

Sample B – PDMS

For the PDMS coating we examined a set of 85 NV centers, out of which 13 showed proton signal via an XY8-10 sequence. Only 2 of these 13 NVs reveal a noticeable signal after an XY8-3 correlation sequence.

The two correlation curves shown in Supplementary Figure 2 show the curve presented in Supplementary Figure 3 of the main text (NV1) and a second curve for a second NV (NV78). As explained above, experiments with this sample suffered from poor collection efficiency and required particularly long integration times, the reason why the second data set was measured only for evolution times $< 20\ \mu s$. Both measured signals are overlaid with the same calculated decay envelope, assuming a slow molecular dynamics of the PDMS molecules as described in the main text (see also Supplementary Note 4 below).

Sample C – Immersion oil

For the immersion oil a set of 83 NV centers was measured. Among them, nine NVs exhibited proton signal after an XY8-10 sequence. Five of those NVs showed an XY8-3 correlation signal. We measured two of those NVs over a longer evolution time window ($> 80 \mu s$, NV6 is the one shown in the main text and related SI notes). As shown in Supplementary Figure 3, we measure virtually identical responses characterized by a fast initial decay and a long-lived tail as highlighted in the main text. Remarkably, the two data sets are so consistent that the same set of parameters (adsorbed layer thickness, translational diffusion constant, and rotational diffusion constant) can be used to describe both observations.

Origin of the signal

Recently it was pointed out that the XY8-N noise spectroscopy is prone to measure spurious effects of higher Larmor frequency harmonics[1]. Especially the 4th harmonic of the ^{13}C nuclei intrinsic in the diamond lattice is spectrally located very close to the proton Larmor frequency, and can be easily misinterpreted as the signal of external protons. Our protocol is immune to this problem because it directly records the proton spin precession during $\tilde{\tau}$, thus leading to resonance spectra at the specific nuclear Larmor frequency[2]. In particular, any residual effect from carbon spins during the XY8-N segments of the correlation protocol would lead to a peak at the carbon spin resonance frequency, which can be easily separated from proton-induced contributions.

Supplementary Note 2

Modeling the correlation signal envelope

The envelope of the correlation signal is described by (see Eq. (2) of the main text)

$$\sum_{i,j} p_{i,j}(\tilde{\tau})\, e^{-\tilde{\tau}/T_2^{i,j}}, \qquad (1)$$

where $p_{i,j}(\tilde{\tau})$ is the conditional probability that the *i*-th nuclear spin of the *j*-th molecule remains in the detection volume over the evolution time $\tilde{\tau}$, and $T_2^{i,j}$ is the transverse relaxation time of said nuclear spin. Here we neglect any additional contribution to the envelope arising from longitudinal relaxation of the NV electron spin (see below Supplementary Note 6). This is a reasonable assumption as typical longitudinal relaxation times are in the order of hundreds of µs, while we focus here on effects on timescales of a few tens of µs, i.e. the time range of our measurements. Before describing how we model the probability and relaxation time in detail, we introduce below the underlying assumptions and simplifications of the presented theory.

First, we assume that the value of the sum in Supplementary Eq. (1) is time independent, i.e. the total number of protons/molecules inside the detection volume stays approximately constant over

time. Therefore the amplitude and decay components of the correlation signal depend solely on the spatial distribution of the molecular mobility with respect to the surface distance. Further, we neglect any hydrodynamic interactions among molecules other than those implicit when assigning a candidate translation or rotation diffusion coefficient.

The molecules are treated as rigid spheres of radius a, and their molecular mobility varies on the length scale of the detection volume. This heterogeneity is described by position dependent diffusion constants $D_{\mathrm{T}}(\vec{r})$ and $D_{\mathrm{R}}(\vec{r})$ for the translational and rotational diffusion respectively, which takes into account the possible presence of adsorbate molecules on the diamond surface. We estimate that the transition between adsorbate and free molecules is sharp and that the latter obey classical diffusion equations, as well as the Stokes-Einstein-(Debye) equations for the diffusion constants

$$D_{\mathrm{T}} = \frac{k_{\mathrm{B}}T}{6\pi\eta a} \quad ; \qquad (2)$$

$$D_{\mathrm{R}} = \frac{k_{\mathrm{B}}T}{8\pi\eta a^3} \quad ; \qquad (3)$$

where k_{B} is the Boltzmann constant, T is the medium temperature, and η is the viscosity of the sample medium.

We further assume that the diffusion heterogeneity is restricted to the z-axis perpendicular to the diamond surface. The rotational diffusion constant can have a finite value at the surface, while the translational diffusion constant goes to zero. These values stay approximately constant up to a distance z_{ads} to the surface, which we define as the thickness of the adsorbate layer. Beyond this layer, the diffusion constants are related to each other via $D_{\mathrm{R}} = \frac{3}{4a^2} D_{\mathrm{T}}$. We assign to both diffusion constants a similar transition profile, which we model as (Supplementary Figure 4a)

$$D(z) = \begin{cases} D_{\mathrm{surface}}, & z \leq z_{\mathrm{ads}} \\ (D_{\mathrm{bulk}} - D_{\mathrm{surface}}) \left(1 - \exp\left(-\frac{z - z_{\mathrm{ads}}}{\zeta}\right)\right) + D_{\mathrm{surface}}, & z > z_{\mathrm{ads}} \end{cases}, \qquad (4)$$

where D_{bulk}, and D_{surface} are the respective values of the diffusion constants in the bulk of the volume and at the surface, and ζ is a transition parameter, which we keep fixed at an empirical value of $\zeta = 0.25\ \text{nm}$. We note that this value is in the same order of magnitude as that found for AFM experiments measuring the transition of surface to bulk fluid dynamics[3]. We find that the exact value of ζ has a negligible impact on the results of the simulation, as long as the transition takes place on a scale small compared to the radial size of the detection volume.

The coherences of the nuclear spin bath characterized by the transverse relaxation time $T_2^{i,j}$ can be calculated using established means known from classical NMR[4]. In the following we will briefly outline the calculation steps of the relaxation time, before describing the model we use to estimate the conditional probability $p_{i,j}(\tilde{\tau})$.

Nuclear spin relaxation time T_2

The nuclear spin dynamics mainly depends on position dependent correlation times of motion τ_i. The only interaction considered among the nuclei is homonuclear dipolar coupling, so that one can distinguish different time regimes by the ratio of the rate of nuclear spin reorientation ($\propto\ \tau_i^{-1}$) versus the dipolar coupling constant ω_D.

Among the interactions responsible for the correlation decay one can distinguish between intramolecular interactions inside a molecule, and intermolecular interactions among spins of different molecules. In the interior of a molecule, the variation of the dipolar coupling between spins arises almost exclusively from the rotation of the molecule (neglecting distance variations due to vibrations), while for the interactions between spins in different molecules their relative translation must be considered.

1H-1H interaction

The homonuclear dipolar coupling between two proton spins is described by the Hamiltonian

$$H_{\text{dip}} = \frac{\mu_0 \hbar^2}{4\pi} \frac{\gamma_{1\text{H}}^2}{r_{\text{HH}}^3} \left(I_1 I_2 - 3\frac{(\vec{r}_{\text{HH}}\, I_1)(\vec{r}_{\text{HH}}\, I_2)}{\vec{r}_{\text{HH}}^2}\right), \qquad (5)$$

where μ_0 is the magnetic constant, $\hbar$ is the reduced Planck constant, $\gamma_{1\mathrm{H}}$ is the gyromagnetic ratio of a proton spin, I_i are the spin vector operators of the i-th proton, and $\vec{r}_{\mathrm{HH}}$ is the distance vector between two proton spins. Squaring Supplementary Eq. (5), averaging over the azimuthal and polar angles, and taking the trace over the spin matrices yields an expression for the rms-dipolar coupling strength

$$\sqrt{\omega_{\mathrm{D}}^2} = \frac{63}{8}\sqrt{\frac{3}{5}}\,\frac{\mu_0\,\hbar}{4\pi}\,\frac{\gamma_{1\mathrm{H}}^2}{r_{\mathrm{HH}}^3}\ , \qquad (6)$$

where we use a representation of $\vec{r}_{\mathrm{HH}}$ in spherical coordinates. In Supplementary Eq. (6) r_{HH} is the average distance to the nearest nuclear neighbor, which is dependent on the proton density ρ and is distributed according to

$$P(r_{\mathrm{HH}}) = 4\pi\rho\, r_{\mathrm{HH}}^2 \exp\!\left(-\frac{4\pi}{3} r_{\mathrm{HH}}^3\rho\right)\ . \qquad (7)$$

This results in an average proton-proton distance of

$$\langle r_{\mathrm{HH}}\rangle \approx \frac{1}{2}\,\rho^{-\frac{1}{3}}\,. \qquad (8)$$

Combining Supplementary Eq. (8) and Supplementary Eq. (6) yields an expression for the average interaction strength

$$\sqrt{\omega_{\mathrm{D}}^2} = 63\sqrt{\frac{3}{5}}\,\frac{\mu_0\hbar}{4\pi}\,\gamma_{1\mathrm{H}}^2\rho. \qquad (9)$$

The relaxation rate of the axial magnetization component for a 2-spin cluster due to their mutual dipolar interaction is calculated via[4]

$$f_{\mathrm{dip}} = \frac{3}{2}\,\frac{\mu_0\hbar}{4\pi}\,\gamma_{1H}^2\,\frac{4\pi^2}{3}\rho\ \ . \qquad (10)$$

Since we assume a constant proton density throughout the detection volume, the average interaction strength and dipolar relaxation rate are therefore position independent. Using a typical

value for the proton density in organic compounds of $\rho = 50\,\mathrm{nm}^{-3}$ for all samples, yields a fluctuation rate of $f_{\mathrm{dip}} \approx 20$ kHz, and average interaction strength of $\sqrt{\omega_{\mathrm{D}}^2} \approx 45$ kHz.

Translational diffusion of 1H spins

The translational diffusion of molecules generates a small fluctuating magnetic field. The fluctuation rate due to translational diffusion is given by

$$f_{\mathrm{trans}}(\vec{r}) = 2D_{\mathrm{T}}(\vec{r}) \left(\frac{1}{2\,a}\right)^2. \qquad (11)$$

Rotational diffusion of 1H spins

For a spherical molecule of radius a rotating in a liquid of viscosity η the rotational fluctuation rate is described by Stokes' law

$$f_{\mathrm{rot}}(\vec{r}) = \frac{3\,k_{\mathrm{B}}T}{4\pi a^3\,\eta(\vec{r})}. \qquad (12)$$

Using the Stokes-Einstein relation (Supplementary Eq. (3)) we find

$$f_{\mathrm{rot}}(\vec{r}) = 6D_{\mathrm{R}}(\vec{r}). \qquad (13)$$

Combined dynamics and relaxation rate

In order to describe the nuclear spin relaxation dynamics of the sample molecules we use an autocorrelation function of the fluctuating magnetic field B(t) experienced by the nuclear spins

$$\langle B(t)B(t')\rangle = \langle B^2\rangle \exp(-\frac{|t-t\prime|}{\tau_{\mathrm{c}}}), \qquad (14)$$

where $\langle B^2\rangle$ is the rms field strength experienced by the protons spins, and τ_{c} is the characteristic correlation time of the magnetic fluctuations, which includes all above mentioned mechanisms, i.e. $\tau_{\mathrm{c}}(\vec{r}) = \left(f_{\mathrm{dip}} + f_{\mathrm{trans}}(\vec{r}) + f_{\mathrm{rot}}(\vec{r})\right)^{-1}$. The spectral density $\tilde{J}(\omega, \tau_{\mathrm{c}})$ is twice the Fourier transform of Supplementary Eq. (14)

$$\tilde{J}(\omega,\tau_{\mathrm{c}}) = 2\langle B^2\rangle \frac{\tau_{\mathrm{c}}}{1+\omega^2\tau_{\mathrm{c}}^2}\,. \tag{15}$$

The normalized spectral density $J(\omega,\tau_{\mathrm{c}})$ is

$$J(\omega,\tau_{\mathrm{c}}) = \frac{\tau_{\mathrm{c}}}{1+\omega^2\tau_{\mathrm{c}}^2}\,. \tag{16}$$

The transverse relaxation is caused by the dephasing of the proton spins due to their mutual dipolar interactions. In the case of intramolecular (homonuclear) dipole-dipole relaxation, the transverse relaxation rate T_2^{-1} between two spin ½ nuclei is given by[4]

$$T_2^{-1}(\vec{r}) = \frac{3}{20}\sqrt{\omega_D^2}^{\,2}\left[3J\big(0,\tau_c(\vec{r})\big) + 5J\big(\omega_L,\tau_c(\vec{r})\big) + 2J\big(2\omega_L,\tau_c(\vec{r})\big)\right], \tag{17}$$

where ω_L is the nuclear Larmor precession frequency, and $\sqrt{\omega_{\mathrm{D}}^2}$ is the average dipole-dipole coupling constant, given by Supplementary Eq. (6).

Probability $p_{i,j}(\tilde{\tau})$

We model the probability $p_{i,j}(\tilde{\tau})$ using a semiquantitative geometric model. Simply put, we draw a 'diffusion sphere' around a starting position $\vec{r}$ with a radius

$$r_{\mathrm{diff}}(\vec{r},\tilde{\tau}) = \sqrt{6\,D_{\mathrm{T}}(\vec{r})\,\tilde{\tau}}, \tag{18}$$

which is the rms-diffusion distance in three dimensions for Brownian motion. We only consider translational diffusion here, and the translational diffusion constant $D_{\mathrm{T}}(\vec{r})$ only depends on the starting position $\vec{r}$ (Supplementary Figure 4a, Supplementary Figure 5a). The conditional probability to find the molecule inside the detection volume (V_{DV}) after the time $\tilde{\tau}$ is then approximately given by the ratio of the overlap between the diffusion volume (V_{diff}) and the detection volume, normalized to the detection volume (Supplementary Figure 5b):

$$p_{i,j}(\tilde{\tau}) = \frac{V_{\mathrm{diff}} \cap V_{\mathrm{DV}}}{V_{\mathrm{DV}}}. \tag{19}$$

The detection volume is simplified as a hemisphere with radius $R_{\mathrm{DV}} = 4\;\mathrm{nm}$, which roughly

translates to an expected volume of (5 nm)³.

The overlap $V_{\mathrm{diff}} \cap V_{\mathrm{DV}}$ can be calculated via the intersection of two spheres

$$V_{\mathrm{intersec}} = \frac{\pi\,(R_{\mathrm{DV}} + r_{\mathrm{diff}} - r)^2 (r^2 + 2r(R_{\mathrm{DV}} + r_{\mathrm{diff}}) - 3(R_{\mathrm{DV}} - r_{\mathrm{diff}})^2)}{12\,r}, \tag{20}$$

where r is the absolute value of the starting position vector $\vec{r}$, and r_{diff} is obtained from Supplementary Eq. (18). The intersection volume will have a negative value if either the diffusion sphere is completely inside the detection volume ($p_{i,j}(\tilde{\tau}) = 1$), or if the diffusion sphere becomes larger than the detection volume. In the latter case the probability $p_{i,j}(\tilde{\tau})$ is determined by the ratio of the diffusion sphere volume to the detection volume.

Since the detection volume is only a hemisphere, we have to subtract any intersection or diffusion volume contributions extending below the surface before calculating the value for the probability. The volume beneath the surface can be calculated as the volume of a sphere cap (V_{cap}). Therefore the volume below the surface can be calculated as

$$V_{\mathrm{below}} = V_{\mathrm{cap}} - V_{\mathrm{piece}}, \tag{21}$$

with $V_{\mathrm{cap}} = \frac{\pi}{3} h^2 (3r_{\mathrm{diff}} - h)$, where $h = r_{\mathrm{diff}} - z$ is the height of the cap, and z is the z-component of the position vector $\vec{r}$. The volume fraction to be subtracted is approximately given by

$$V_{\mathrm{piece}} \approx \frac{c}{2b} V_{\mathrm{cap}}, \tag{22}$$

where $b = \sqrt{r_{\mathrm{diff}}^2 - z^2}$ is the radius of the circular base of the cap volume, and $c = a + b - R_{\mathrm{DV}} = \sqrt{r^2 - z^2} + \sqrt{r_{\mathrm{diff}}^2 - z^2} - R_{\mathrm{DV}}$.

Supplementary Note 3

Monte Carlo (MC) simulation of the molecular dynamics in the detection volume and comparison to the experimental data

For the simulation we pick random starting positions $\vec{r}$ inside the detection volume, then calculate the probability to remain within this volume for varying $\tilde{\tau}$ as described above, i.e. varying the radii of the diffusion sphere. For each $\tilde{\tau}$ we calculate an average correlation time $\langle T_2^{(i,j)} \rangle$ between the highest and the lowest z-value in the diffusion sphere. The probability is then multiplied by the NMR decay $\exp(-\tilde{\tau}/\langle T_2^{(i,j)} \rangle)$. We do an ensemble average over multiple starting positions to get the envelope of the correlation signal. We vary the values of the adsorption layer thickness z_{ads}, the rotational diffusion constant at the surface $D_{\mathrm{R,surface}}$, and the translational diffusion constant in the bulk $D_{\mathrm{T,bulk}}$, and overlay the results with the experimental data to obtain quantitative estimates of the molecular mobility in the samples.

We find good qualitative agreement between the simulation and the experimental data. For Sample C (immersion oil, Supplementary Figure 6) the model manages to reproduce both the initial fast decay (attributed to the molecules diffusing outside the detection volume), and the long lasting signal tail (described by the NMR decay of the molecules inside the adsorption layer). The 'amplitude' of the long lasting decay is set by the thickness of the adsorption layer (Supplementary Figure 5d), from which a value of 1.5 nm is estimated. This result is in good agreement with prior experimental observations[5,6], and would correspond to approximately 1-2 layers of molecular adsorbates on the surface. The translational diffusion constant mainly influences the fast initial drop of the signal, and we find best agreement for a value of $D_{\mathrm{T,bulk}} \sim 0.3$ nm^2/μs (Supplementary Fig. 6). We note that this is in the order of magnitude that one would expect from Supplementary Eq. (2), which yields a value of $D_{\mathrm{T,bulk}} \sim 1.0$ nm^2/μs, corresponding to a molecule of radius a = 0.5 nm, a medium of viscosity $\eta = 437$ mPa.s (as listed in the compound datasheet), and room temperature conditions. The deviations could be readily explained by varying the molecule radius a, as the

immersion oil contains various different molecules of different chain length etc., which are treated identically in the simulations.

Our model also manages to describe the decay times of the purely exponential signal envelopes for the solid polymers quite well. Here we assume no translational diffusion throughout the detection volume, i.e. the whole volume is treated as an 'adsorption layer'. We then vary the value of the rotational diffusion constant $D_{R,surface}$ until we obtain a reasonable match with the experimental data.

In the case of Merckoglas (sample A) we can only reproduce the signal shape by removing the rotational diffusion as well, i.e. molecules remain static over the course of the measurement. In this case the probability $p_{i,j}(\tilde{\tau})$ becomes unity, and the signal decay is mainly governed by NMR relaxation due to inter- and intramolecular dipolar interactions, yielding a fast single exponential decay (Supplementary Figure 7a) as expected for a solid.

For PDMS the correlation signal also decays exponentially but the decay time (of order ~30 μs) is found to be longer than anticipated for an ensemble of static, dipolarly-coupled protons (at least assuming a standard proton density of ρ = 50 nm^{-3}). We can reproduce the observed behavior by assuming a rotational diffusion constant of $D_{R,surface}$ = 0.001 rad^2/μs throughout the detection volume. The latter is supported by prior NMR experiments showing that PDMS can exhibit significant molecular dynamics at room temperature, thus leading to partial motional narrowing[7]. The self-diffusion depends on the PDMS chain length/molecular weight, i.e., on the mixing ratio of the components. Even if partly, this enhanced mobility can be the reason of the line narrowing captured in the non-negligible value of the rotational diffusion constant in the simulation. For completeness, we note that the observed data set can also be reproduced by a static ensemble of protons with a ~20% lower density than normal (40 nm^{-3} as opposed to 50 nm^{-3}, lighter green trace in Supplementary Figure 7b). However, in light of the experimental evidence mentioned above, this alternative seems less realistic.

Supplementary Note 4

Model precision and accuracy

As described above the key parameters in our model are the bulk translational diffusion coefficient $D_{\mathrm{T,bulk}}$ (which governs the initial signal decay for a liquid sample), the rotational diffusion coefficient in the semi-mobile adsorption layer $D_{\mathrm{R,surface}}$ (describing the decay time of the long lasting signal tail), and the thickness of the adsorption layer z_{ads} (which sets the amplitude of this long signal component). Supplementary Figure 6 schematically visualizes the role of each parameter on the resulting decay envelope.

As shown in Supplementary Figure 8 for Sample C, we find that the modeled response of NV 6 is quite sensitive to the values assigned to these parameters. For example, Supplementary Figure 8a shows the initial decay of the correlation signal due to the translational diffusion of the sample molecules for varying translational diffusion coefficients $D_{\mathrm{T,bulk}}$. Here the values for the rotational diffusion inside the semi-mobile layer ($D_{\mathrm{R,surface}} = 0.05\ \mathrm{rad^2/\mu s}$) and the adsorption layer thickness ($\mathrm{z_{ads}} = 1.5\ \mathrm{nm}$) are kept constant. One can qualitatively estimate a confidence interval (light blue shaded area) of $D_{\mathrm{T,bulk}} = [0.2\frac{\mathrm{nm^2}}{\mathrm{\mu s}}, 0.4\frac{\mathrm{nm^2}}{\mathrm{\mu s}}]$ for which the model parameters reproduce the envelope shape to a comparable extent. Beyond this interval the calculated response notably departs from the measurement, allowing us to distinguish changes of the diffusion coefficient within $\sim 30\%$.

Supplementary Figure 8b shows the long decay component of the correlation signal for varying rotational diffusion coefficients $D_{\mathrm{R,surface}}$. Here we keep the values for the translational diffusion in the bulk layer ($D_{\mathrm{T,bulk}} = 0.3\ \mathrm{nm^2/\mu s}$) and the adsorption layer thickness ($\mathrm{z_{ads}} = 1.5\ \mathrm{nm}$) constant. Over the measured proton evolution time we find that the correlation amplitude stays approximately constant, with no distinct signal decay. Therefore we can only estimate a lower bound for the rotational diffusion coefficient for which we can reproduce this behavior. We find that this

signal shape is reproduced by rotational diffusion coefficients larger than $D_{\mathrm{R,surface}} = 0.05\,\frac{\mathrm{rad}^2}{\mu\mathrm{s}}$, and that a notable deviation from the data can be observed for coefficients below $D_{\mathrm{R,surface}} = 0.02\,\frac{\mathrm{rad}^2}{\mu\mathrm{s}}$. Therefore within the measurement region the model can distinguish changes below $0.05\,\frac{\mathrm{rad}^2}{\mu\mathrm{s}}$ with a precision of $\sim 60\%$.

Supplementary Figure 8c shows the variation in the amplitude of the long decay component of the correlation signal for varying adsorption layer thicknesses. In this case the translational diffusion in the bulk layer ($D_{\mathrm{T,bulk}} = 0.3\ \mathrm{nm}^2/\mu\mathrm{s}$) and the rotational diffusion in the adsorption layer ($D_{\mathrm{R,surface}} = 0.05\,\frac{\mathrm{rad}^2}{\mu\mathrm{s}}$) are kept constant. We can estimate a confidence interval of $\mathrm{z_{ads}} = [1.25\ \mathrm{nm}, 1.75\ \mathrm{nm}]$, which results in comparable decay responses corresponding to a precision of $\sim 17\%$.

The relatively good precision of our model in discriminating between different numerical values of the fit parameters must be distinguished from the model absolute accuracy which, in light of the various simplifications (see Supplementary Note 3), should be considered only moderate. For example, one of the underlying assumptions is that the sample molecules are spherical and that they diffuse according to a free Brownian motion. The effect of anisotropic diffusion due to molecular non-sphericity is difficult to estimate. We can, however, qualitatively examine the effects of non-Brownian motion by considering the case of anomalous diffusion. In this case, the diffusion radius is given by

$$r_{\mathrm{diff}}^{anomalous}(\vec{r},\tilde{\tau}) = \sqrt{\Gamma\, D_{\mathrm{T}}(\vec{r})\, \tilde{\tau}^{\alpha}}, \qquad (23)$$

where Γ is a scaling factor, and α characterizes the diffusion process. In the case of a typical (Brownian) diffusion $\alpha = 1$, while $\alpha > 1$ is often associated with super-diffusion processes (e.g., due to cellular transport processes).

We set $\Gamma = 6$ (corresponding to 3D Brownian diffusion) since the exact value only affects the

magnitude of the diffusion constant but not the decay envelope. Supplementary Figure 8d shows the transition from Brownian diffusion to super-diffusion as we increase the exponent α, while keeping the other values constant ($D_{\mathrm{T,bulk}} = 0.3\ \mathrm{nm}^2/\mu\mathrm{s}$, $D_{\mathrm{R,surface}} = 0.05\frac{\mathrm{rad}^2}{\mu\mathrm{s}}$, $\mathrm{z_{ads}} = 1.5\ \mathrm{nm}$).

The change to super-diffusion leads to a faster signal decay, while maintaining an overall shape qualitatively similar to free diffusion. This leads to a certain ambiguity in determining the diffusion regime, as the faster signal decay can be partly compensated by reducing the value of Γ and/or $D_{\mathrm{T,bulk}}$.

Supplementary Note 5

Role of NV spin-lattice relaxation

The observation of a correlation signal depends on the NV ability to store the phase picked up during the first interrogation segment throughout the evolution interval $\tilde{\tau}$. A question of interest is, therefore, whether NV spin-lattice relaxation T_{1NV} must be taken into account in our modeling.

While we did not specifically determine T_{1NV} for the NVs we used in our correlation measurements, observations from virtually identical NVs are presented in Supplementary Figure 9. The plotted data points correspond to the difference between pump-probe and inversion-recovery protocols. Out of the 25 NVs we studied, we determine an average spin-lattice relaxation time of ~350 µs with a maximum dispersion between T_{1NV} values of about 100 µs. These relaxation times are substantially longer than the time constants describing the decay of the correlation signal in the solid samples (~20 µs and ~30 µs for Samples A and B, respectively), confirming the above conclusion that NV relaxation can be ignored in these two cases. Sample C, on the other hand, deserves some special consideration: The first segment of the correlation signal (governed by molecular diffusion and decaying on a time scale of $T_{corr}^{(short)}{\sim}15$ µs) is clearly insensitive to NV spin lattice relaxation, meaning that no corrections are required in our numerical estimates of the bulk diffusion constant

$D_{\mathrm{T,bulk}}$ and the adsorbed layer thickness z_{ads}. This is, however, not the case for the longer-lived segment of the correlation signal, which decays on a time scale $T_{corr}^{(long)}\sim 250$ μs comparable to T_{1NV} (Supplementary Figure 10b). Therefore, our estimate of the rotational diffusional constant in the adsorbed layer $D_{R,ads}$ must be interpreted as a lower bound.

Comparison with bulk NMR data

Relevant information on the sample dynamics near the diamond surface can be gained by comparing the measured correlation signals with bulk ^{1}H NMR data. Supplementary Figure 10 shows the NMR proton signal for Sample A (Merckoglas) and Sample B (PDMS) upon application of a π/2-pulse at 600 MHz (corresponding to a 14.1 T magnetic field). In both cases we find that the NV-detected correlation signals (Supplementary Figs. 1 and 2) are shorter-lived than their inductively detected counterparts (^{1}H NMR FIDs in Supplementary Figs. 10a and 10c). The difference is starker in the case of Sample B, where the nuclear spin coherence time is seen to persist beyond 0.5 ms. We interpret these differences as a manifestation of the singular molecular dynamics near the diamond surface, where motion is likely more restricted than in the bulk.

Indirect support for this idea is provided by the results of Supplementary Figs. 10b and 10d where we expose the importance of molecular packing in the dynamics of both Sample A and Sample B: In the first case (Supplementary Figure 10b), we let a sample of 'as-purchased' Merckoglas dry to form a solid crust, which we then analyze via inductive NMR. This system differs from the one used in Supplementary Fig. 10a only in its preparation protocol: Unlike the practice in our NV experiments—where Mercoglas is dissolved in toluene to form a 1:1 solution prior to gluing the diamond crystal to the sample holder—no solvent was added. The result is an ^{1}H FID substantially longer than that in Supplementary Figure 10a, the increased mobility likely originating from the reduced number of interstitial molecules after solvent evaporation. Interestingly, we observe the converse effect in Sample B where we shorten the FID duration by bringing the ratio between PDMS and the curing agent to a value lower than that used for our NV experiments. The latter leads to a more rigid form

of the resulting polymer and thus a shorter-lived proton FID.

For completeness, Supplementary Figure 11 presents ^{1}H NMR data corresponding to Sample C (immersion oil), where we find a proton spin transverse relaxation time of order 25 ms, limited by field inhomogeneity (an FID from acetone is included as a reference).

Supplementary References

[1] Loretz, M. et. al., Spurious harmonic response of multipulse quantum sensing sequences. *Phys. Rev. X* **5**, 021009 (2015).

[2] Laraoui, A. et. al., High-resolution correlation spectroscopy of 13C spins near a nitrogen-vacancy centre in diamond. *Nature Commun.* **4,** 1651 (2013).

[3] Ortiz-Young, D., Chiu, H.-C., Kim, S., Voitchovsky, K. & Riedo, E. The interplay between apparent viscosity and wettability in nanoconfined water. *Nature Commun.* **4,** 2482 (2013).

[4] Levitt, M. H. *Spin Dynamics: Basics Of Nuclear Magnetic Resonance* (John Wiley & Sons, Hoboken, 2008).

[5] Rugar, D. et. al., Proton magnetic resonance imaging using a nitrogen-vacancy spin sensor. *Nature Nanotech.* 10, 120-124 (2014).

[6] Häberle, T., Schmid-Lorch, D., Reinhard, F. & Wrachtrup, J. Nanoscale nuclear magnetic imaging with chemical contrast. *Nature Nanotech.* 10, 125-128 (2015).

[7] Litvinov, V. M. & Spiess, H. W. ^{2}H NMR study of molecular motions in polydimethylsiloxane and its mixtures with aerosils. *Die Makromolekulare Chemie* **192,** 3005-3019 (1991).
